# Incidence and risk factors for retinopathy of prematurity in a Brazilian reference service

**DOI:** 10.1590/1516-3180.2014.1322544

**Published:** 2014-04-01

**Authors:** Eduardo Gonçalves, Luciano Sólia Násser, Daniella Reis Martelli, Isadora Ramos Alkmim, Thalita Veloso Mourão, Antônio Prates Caldeira, Hercílio Martelli-Júnior

**Affiliations:** I MD. Doctoral Student and Professor, Postgraduate Health Science Program, Universidade Estadual de Montes Claros (Unimontes), and Faculdades Integradas Pitágoras (FIPMoc), Montes Claros, Minas Gerais, Brazil; II MD. Master's Student, Postgraduate Health Science Program, Universidade Estadual de Montes Claros (Unimontes), Montes Claros, Minas Gerais, Brazil; III MD. Doctoral Student and Professor, Postgraduate Health Science Program, Universidade Estadual de Montes Claros (Unimontes), Montes Claros, Minas Gerais, Brazil; IV Medical Student, Universidade Estadual de Montes Claros (Unimontes), Montes Claros, Minas Gerais, Brazil; V Medical Student, Faculdades Integradas Pitágoras (FIPMoc), Montes Claros, Minas Gerais, Brazil; VI MD, PhD. Professor, Postgraduate Health Science Program, Universidade Estadual de Montes Claros (Unimontes), Montes Claros, Minas Gerais, Brazil

**Keywords:** Retinopathy of prematurity, Incidence, Infant, premature, Risk factors, Gestational age, Retinopatia da prematuridade, Incidência, Prematuro, Fatores de risco, Idade gestacional

## Abstract

**CONTEXT AND OBJECTIVE:**

Retinopathy of prematurity (ROP) is a known cause of blindness in which diagnosis and timely treatment can prevent serious harm to the child. This study aimed to evaluate the incidence of ROP and its association with known risk factors.

**DESIGN AND SETTING:**

Longitudinal incidence study in the neonatal intensive care unit (NICU) of Universidade Estadual de Montes Claros.

**METHODS:**

Newborns admitted to the NICU with gestational age less than 32 weeks and/or birth weight less than 1,500 grams, were followed up over a two-year period. The assessment and diagnosis of ROP were defined in accordance with a national protocol. The chi-square test or Fisher's exact test were used to determine associations between independent variables and ROP. Analysis on the independent effect of the variables on the results was performed using multiple logistic regression.

**RESULTS:**

The incidence of ROP was 44.5% (95% confidence interval, CI = 35.6-46.1) in the study population. The risk factors associated with the risk of developing the disease were: birth weight less than 1,000 grams (odds ratio, OR = 4.14; 95% CI = 1.34-12.77); gestational age less than 30 weeks (OR = 6.69; 95% CI = 2.10-21.31); use of blood derivatives (OR = 4.14; 95% CI = 2.99-8.99); and presence of sepsis (OR = 1.99; 95% CI = 1.45-2.40).

**CONCLUSIONS:**

The incidence of ROP was higher than that found in the literature. The main risk factors were related to extreme prematurity.

**CONTEXTO E OBJETIVO:**

A retinopatia da prematuridade (ROP) é causa conhecida de cegueira e diagnóstico e tratamento oportunos podem evitar graves danos à criança. Este estudo objetivou avaliar a incidência da ROP e sua associação com fatores de risco conhecidos.

## Abstract (pt)

**TIPO E LOCAL DE ESTUDO:**

Estudo longitudinal de incidência na Unidade de Terapia Intensiva Neonatal (UTIN) da Universidade Estadual de Montes Claros.

**MÉTODOS:**

Foram acompanhados neonatos admitidos na UTIN, com idade gestacional menor que 32 semanas e/ou peso ao nascimento inferior a 1.500 gramas, por um período de dois anos. Avaliação e diagnóstico de ROP foram definidos conforme protocolo nacional. Utilizou-se o teste qui-quadrado ou teste exato de Fisher para determinar a associação entre as variáveis independentes e ROP. A análise do efeito independente das variáveis sobre o resultado (ROP) foi realizada por meio de regressão logística múltipla.

**RESULTADOS:**

A incidência de ROP foi de 44,5% (intervalo de confiança, IC 95% = 35,6-46,1) entre a população estudada. Os fatores de risco pesquisados que apresentaram relação de risco para o desenvolvimento da doença foram: peso de nascimento menor que 1000 gramas (*odds ratio*, OR = 4,14; IC 95% = 1,34;12,77), idade gestacional menor que 30 semanas (OR = 6,69; IC 95% = 2,10;21,31), uso de hemoderivados (OR = 4,14; IC 95% = 2,99;8,99) e presença de sepse (OR = 1,99; IC 95% = 1,45;2,40).

**CONCLUSÃO:**

A incidência de ROP foi maior que a encontrada na literatura. Os principais fatores de risco estão relacionados à prematuridade extrema.

## INTRODUCTION

Retinopathy of prematurity (ROP) is a vasoproliferative eye disease of multifactorial etiology that affects the retinal vascularization of premature infants.^1^ ^,^ ^2^ The importance of ROP lies in its frequency and in prevention of blindness due to this condition, given that, once diagnosed and treated, it is unlikely to develop into complete loss of vision.^3^ ^-^ ^6^ The incidence of blindness varies between countries, and it is influenced by the level of perinatal care and the existence of screening programs for early diagnosis.^7^

In the United States, around 0.12% of all live births develop ROP, or one case for every 820 newborns, and there are an estimated 300 new cases of blindness annually due to ROP in that country.^3^ In Brazil, studies have shown increased numbers of ROP cases, especially in large centers.^7^ It is known that ROP is multifactorial, due to the immaturity of the preterm retina,^3^ ^,^ ^8^ and that the risk factors are: prematurity, low birth weight, oxygen therapy, intracranial hemorrhage and persistent ductus arteriosus, among others.^8^ ^-^ ^10^

## OBJECTIVE

The aim of this study was to evaluate the incidence of ROP at a Brazilian reference service, along with the main known risk factors for this important condition and its association with morbidity.

## METHODS 

This was a prospective longitudinal incidence study and it was approved by the Ethics Committee for Institutional Research of Montes Claros State University (Universidade Estadual de Montes Claros, Unimontes), in Minas Gerais, Brazil (protocol 2013/10).

In this study, a convenience sample limited to a period of time was used. The study included preterm infants who had been admitted to the neonatal intensive care unit (NICU) of the Unimontes university hospital, between May 2009 and April 2011. The inclusion criteria were that the birth weight should be ≤ 1,500 g and/or the gestational age should be ≤ 32 weeks, with survival at least until the sixth week of life;^6^ and that the infants were treated as outpatients for follow-up care in the eye clinic. The study excluded infants born with ocular malformation or ocular genetic alterations and those who did not survive past the sixth week of life. 

There was no sample calculation. Over the period from May 2009 to April 2011, 124 patients who met the inclusion criteria were admitted. However, 12 patients died and two did not return to the clinic for monitoring. The eye examination took place in the sixth week of life, or between 32 and 36 weeks of corrected gestational age. It consisted of using a binocular indirect ophthalmoscope (BIO) under pupil dilation produced by the eye drops 0.5% tropicamide (Midriacyl, Alcon) and 2.5% phenylephrine (Fenilefrina, Allergan), which were applied twice with an interval of ten minutes. The test was completed one hour afterwards and, when necessary, anesthetic eye drops (Anestalcon, Alcon) were also used. A 28-diopter lens (Nikon, Melville, NY, USA) and lid speculum (Alfonso Eye Speculum, Storz, Bausch & Lomb Inc., San Dimas, CA, USA) were used. Scleral indentation was used when necessary.

Children who did not present any degree of retinopathy of prematurity were not considered to be ROP patients. Children who had some degree of ROP were considered to have ROP. The degree of retinopathy assigned to each patient was that of the severest degree seen in the eyes of the infant under examination.^6^

The evaluations were repeated periodically, in accordance with the procedures laid out in the Brazilian Examination and Treatment Guidelines for individuals with ROP.^6^ The ophthalmological examinations were always performed by the same ophthalmologist, who had had specialized retinal training, in the neonatology center. The ophthalmologist did not have any prior knowledge of the medical histories of any of the patients. The examinations were conducted at the neonatal unit and the patients were monitored until reaching 42 weeks of corrected gestational age, or until complete retinal stabilization had been achieved. 

The ophthalmological monitoring of the patients was performed based on the stage of the disease. Patients with incomplete vascularization of the retina were monitored at intervals of one to three weeks until vascularization had been completed. Infants with ROP of degrees I, II or III^4^ (excluding threshold disease) underwent weekly monitoring until complete vascularization of the retina had been achieved. Those with threshold disease underwent treatment for retinal photo-coagulation or cryotherapy. Infants with ROP of degree IV underwent evaluation of scleral explant with or without associated photo-coagulation or cryotherapy or posterior vitrectomy. For infants with ROP of degree V, possible surgical treatment needed to be discussed. Because of technical limitations, patients requiring treatment by means of photo-coagulation, cryotherapy or another surgical procedure were referred to another unit of the hospital.

The assessed risk factors for ROP were categorized based on the characteristics of the newborns, the types of therapies used and the diseases detected. The characteristics of the newborns were evaluated according to gestational age (calculated by the unit's neonatologist using data on the last menstrual period as the first option, followed by ultrasound data and data obtained using the New Ballard method),^11^ birth weight, presence of multiple gestations, Apgar score in the first and fifth minutes and the severity score, SNAPPE II (Score for Neonatal Acute Physiology Perinatal Extension).^12^ ^,^ ^13^

In relation to the therapies used in this study, the following factors were taken into consideration: maximum fraction of inspired oxygen (FiO_2_) level; use of oxygen therapy by means of continuous positive airway pressure (CPAP) or mechanical ventilation; use of indomethacin; use of a surfactant, aminophylline or caffeine; phototherapy use; blood transfusions; use of diuretics; and corticosteroid use. 

For diseases detected during hospitalization, presence of the following was recorded: initial respiratory distress, bronchopulmonary dysplasia (use of oxygen for 28 days or more), sepsis (clinical or laboratorial diagnosis), patent ductus arteriosus (diagnosis by Doppler echocardiography) and intracranial hemorrhage (transfontanellar ultrasound between five and ten days of life).

For statistical analysis, the study population was classified as individuals with ROP and those without ROP, i.e. patients who did not show the illness. The descriptive analysis was performed using absolute numbers and percentages of the qualitative variables and of the central trend averages: means and their respective standard deviations (SD) of the quantitative variables. In further analysis, all the variables were dichotomized.

In the univariate analysis, the chi-square test and the Fisher exact test were used to determine any associations between the independent variables and the outcome (ROP). The magnitude of the effect of the risk factors on the outcome was expressed as the odds ratio (OR), with its respective 95% confidence interval (CI). The analysis on the independent effects of the intervening variables on the outcome (ROP) was performed by means of multiple logistic regression, using forward modeling. All variables that showed P < 0.25 in the association test were included in the modeling process. To build the database and to do the statistical analysis, the Statistical Package for the Social Sciences (SPSS) software (SPSS 18.0 for Windows, SPSS Inc., Chicago, IL, USA) and the Epi Info software (CDC Epi Info 3.5.4, Atlanta, Georgia, USA) were used.

## RESULTS

Between May 2009 and April 2011, 124 children weighing less than 1,500 grams were born, of which 12 died before the first evaluation and 2 did not return for examination after being discharged. Thus, 110 infants were evaluated. The population studied was classified as individuals with ROP, i.e. those who showed some degree of retinopathy of prematurity, and individuals without ROP, i.e. those who did not show retinopathy of prematurity. Among these 110 infants, the prevalence of ROP was 44.5% (95% CI = 35.6-46.1). Considering all the births that took place in the department during the study period, the incidence of ROP was 1.1%. Stage I ROP was the form with the highest incidence among these infants (Table 1), affecting 19 children (17.3% of those with ROP), followed by stage III with 16 children (14.5%) and stage II with 14 children (12.7%). There were no patients with stages IV or V. Two premature infants (1.8%) needed laser treatment because they showed threshold disease. The average weights and gestational ages of all the patients are shown in Table 2.

**Table 1.** Incidence of retinopathy of prematurity (ROP) and disease stag

ROP stage	Incidence	
	n	%
Without ROP	61	55.5
ROP	49	44.5
Total	110	100
I	19	17.3
II	14	12.7
III	16	14.5
IV	0	0
V	0	0
Total	49	44.5

**Table 2.** Means and standard deviations of the birth weight and gestational age risk factors of the different stages of retinopathy of prematurity (ROP

Risk factors	Without ROP (n = 61)	ROP I (n = 19)	ROP II (n = 14)	ROP III (n = 16)	P-value
Weight (grams)	1153.43 ± 241.48	1012.13 ± 183.70	1097.30 ± 213.43	814.35 ± 159.39	0.004
Gestational age (weeks)	30.24 ± 2.58	28.39 ± 2.77	27.96 ± 3.01	26.32 ± 1.69	< 0.001

In the study population, 49.1% of the patients were male. Seventy-four percent of the mothers reported that they had attended prenatal care consultations, 72% presented some complications during pregnancy and 43% had some complications during childbirth. The most common form of delivery was cesarean (65.5%). The SNAPPE II score was obtained for all the preterm infants after they had reached an average of 12 hours of life, ranging from 0 to 88. About half of the population studied was small for the gestational age (49.8%).

Among the risk factors studied, the following correlated significantly (P < 0.05) with the development of ROP, in univariate analysis: gestational age less than 30 weeks, Apgar scores at the first and fifth minute less than 7, SNAPPE II score less than 12, use of blood transfusions, use of diuretics, use of aminophylline or caffeine, use of surfactants, presence of sepsis and presence of bronchopulmonary dysplasia (Table 3).

**Table 3.** Risk factors for development of retinopathy of prematurity (ROP) in infants with weight ≤ 1,500 grams and/or gestational age ≤ 32 weeks; univariate analysi

Risk factors	With ROP (n = 49)	Without ROP (n = 61)	P-value	OR (CI)
	n (%)	n (%)		
Male gender	22 (44.9)	32 (52.5)	0.430	0.74 (0.35-1.57)
Gestational age less than 30 weeks	36 (73.5)	16 (26.2)	< 0.001	7.79 (3.32-18.28)
Birth weight less than 1,000 grams	14 (28.6)	10 (16.4)	0.124	2.04 (0.81-5.11)
Presence of multiple pregnancies	5 (10.2)	4 (6.6)	0.508	0.62 (0.16-2.44)
Use of prenatal corticosteroids	13 (26.5)	10 (16.4)	0.194	1.84 (0.73-4.66)
Apgar score at one minute less than 7	38 (77.6)	35 (57.4)	0.026	2.57 (1.11-5.95)
Apgar score at five minutes less than 7	17 (34.7)	9 (14.8)	0.014	3.07 (1.22-7.70)
Initial respiratory discomfort	46 (93.9)	58 (95.1)	1.000	0.79 (0.15-4.12)
SNAPPE II score less than 12	34 (69.4)	21 (34.4)	< 0.001	4.32 (1.93-9.66)
Oxygen therapy using headpiece (HOOD) for more than 5 days	12 (24.5)	10 (16.4)	0.290	1.65 (0.65-4.23)
Oxygen therapy using CPAP for more than 5 days	13 (26.5)	8 (13.1)	0.075	2.39 (0.90-6.36)
Oxygen therapy using mechanical ventilation for more than 5 days	29 (59.2)	21 (34.4)	0.001	2.76 (1.27-6.01)
Use of blood transfusions	27 (55.1)	13 (21.3)	< 0.001	4.53 (1.97-10.41)
Use of diuretics	30 (61.2)	20 (32.8)	0.003	3.24 (1.48-7.09)
Use of indomethacin	10 (20.4)	8 (13.1)	0.304	1.69 (0.61-4.69)
Use of aminophylline/caffeine	36 (73.5)	33 (54.1)	0.037	2.35 (1.05-5.28)
Use of surfactant	29 (59.2)	20 (32.8)	0.006	2.97 (1.36-6.49)
Presence of sepsis	48 (98.0)	50 (82.0)	0.001	1.98 (1.63-2.41)
Presence of bronchopulmonary dysplasia	26 (53.1)	15 (24.6)	0.002	3.47 (1.54-7.78)
Presence of intracranial hemorrhage	10 (20.4)	10 (16.4)	0.589	1.31 (0.49-3.45)
Presence of patent ductus arteriosus (diagnosed using Doppler echocardiography)	14 (28.6)	13 (21.3)	0.379	1.48 (0.62-3.53)
Use of phototherapy	45 (91.8)	60 (98.4)	0.170	1.10 (0.02-1.73)

OR = odds ratio

CI = confidence interval

CPAP = continuous positive airway pressure

**SNAPPE:** = Score for Neonatal Acute Physiology and SNAP Perinatal Extension

The multivariate analysis using hierarchical logistic regression (the forward method) is represented in Table 4. All of the variables that showed P < 0.25 in the association test were included in the modeling process. Among the risk factors investigated, we found that the following showed a risk of developing ROP: birth weight less than 1,000 grams (OR = 4.14; 95% CI = 1.34-12.77); gestational age less than 30 weeks (OR = 6.69; 95% CI = 2.10-21.31); use of blood transfusions (OR = 4.14; 95% CI = 2.99-8.99); and presence of sepsis (OR = 1.99; 95% CI = 1.45-2.40). There was no verified association with any risk factors, except for the significant trend of the gestational age factor (P = 0.06). 

**Table 4.** Risk factors for retinopathy of prematurity (ROP) development in infants with weight < 1,500 gram

Risk factors	OR^*^	95% CI^†^	P-value
Gestational age less than 30 weeks	6.69	2.10-21.31	< 0.001
Birth weight less than 1,000 grams	4.14	1.34-12.77	0.014
Use of prenatal corticosteroids	1.61	0.45-5.79	0.464
Apgar score at one minute less than 7	1.71	0.59-5.00	0.319
Apgar score at five minutes less than 7	1.32	0.36-4.82	0.670
SNAPPE II score less than 12	1.91	0.62-5.88	0.260
Oxygen therapy using CPAP for more than 5 days	0.92	0.26-3.28	0.899
Oxygen therapy using mechanical ventilation for more than 5 days	0.79	0.21-2.92	0.720
Use of blood transfusions	4.14	2.99-8.99	0.012
Use of diuretics	1.02	0.24-4.55	0.956
Use of aminophylline/caffeine	0.64	0.17-2.38	0.500
Use of surfactant	1.25	0.39-3.96	0.701
Presence of sepsis	1.99	1.45-2.40	< 0.001
Presence of bronchopulmonary dysplasia	1.92	0.68-5.46	0.219
Use of phototherapy	0.27	0.69-1.07	0.062

*OR = odds ratio

†CI = confidence interval

**SNAPPE:** = Score for Neonatal Acute Physiology and SNAP Perinatal Extension

CPAP = continuous positive airway pressure

## DISCUSSION

Over the period of this study, 124 infants with a birth weight of < 1,500 grams were born; however, only 110 entered our department. Twelve children with birth weight < 1,500 grams died (10.9%) and two children (1.8%) did not return to the clinic for follow-up. Thus, the mortality rate was much lower than the national mortality rate of 60% for children born at this weight.^13^

The Cryotherapy for Retinopathy of Prematurity (CRYO-ROP) study showed an incidence of 65.8%.^16^ However, the weight criterion required for inclusion in the study was lower than that of the present study (weight < 1,250 grams). Another important American study, the Early Treatment for Retinopathy of Prematurity Study (ETROP), also included infants with weights of less than 1,250 grams and showed an incidence of 68%.^17^

Although gestational age can sometimes be hard to pinpoint as it is often imprecise and sometimes unknown, gestational age and birth weight were still used as the criteria for this study. These criteria were set because the Brazilian guidelines for screening ROP, published in 2007,^14^ define the inclusion criteria as birth weight < 1,500 g and/or a gestational age < 32 weeks. Additionally, low gestational age and low birth weight have been associated with and have the same consequence as immature retinal tissue.^9^ ^,^ ^18^ ^,^ ^19^ The results from these studies showed that both gestational age and birth weight were associated with ROP. 

Several studies in the literature have shown that the lower the birth weight and the lower the gestational age are, the higher the chance of developing ROP also is.^20^ Moreover, lower birth weight and lower gestational age are associated with the development of more serious forms of ROP. In the current study, stage I of the disease was the most frequently observed form, accounting for 17.3% of the cases when all infants with birth weight < 1,500 grams are included. This proportion rises to 25.8% if only the infants with birth weight < 1,250 grams are included. These findings were not reported in the CRYO-ROP or ETROP studies, which showed higher rates of the severer forms of ROP in infants with lower birth weights and in those with lower gestational age. By analyzing the average birth weight and gestational age among individuals with varying stages of ROP in the present study, it was noted that there was a statistically significant difference between the preterm infants with stage III ROP and those without ROP, thus confirming the association between the immature retina and the ROP stage. 

Just 1.8% of the premature infants in the present study were treated. This small number can be explained by the decrease in stage III cases. It has been questioned whether there is any association between the severity of the SNAPPE II score and retinopathy of prematurity.^14^ The present study showed that although a significant association was observed in univariate analysis, it did not remain in the final model of the multivariate analysis.

The use of oxygen therapy in this study was evaluated in terms of number of days and form of administration. After multivariate analysis, none of the forms of oxygen administration (mechanical ventilation, CPAP or HOOD) showed any statistical association with ROP. Other studies have confirmed the association between the risk of ROP and the use of mechanical ventilation^21^ and CPAP. Oxygenation, regardless of the form of use, has been implicated in causing ROP.^22^ The less mature the preterm infant is, the greater the need for mechanical ventilation and CPAP is, which explains this finding. 

The treatment for bronchopulmonary dysplasia includes use of diuretics and corticosteroids. The present study showed that there was an association between the risk of ROP and the use of diuretics, but only in univariate analysis. In the multicenter STOP-ROP study,^23^ it was found that supplemental use of oxygen therapy for preterm infants with pre-threshold disease increased the risk of chronic pulmonary diseases and also increased the use of diuretics and length of hospital stay. Use of prenatal corticosteroids did not show any statistical impact on development of ROP, which has not been shown in other studies.^24^

The presence of sepsis in our study was considered to be a risk factor for ROP and showed significance. In the literature, this association has already been described.^25^ A study by Chen et al. showed that sepsis can be considered to be an important risk factor for ROP, in screening for preterm infants weighing between 1,501 and 2,000 grams.^26^

The presence of blood transfusions was another risk factor that showed a significant relationship between the groups. This finding has also been reported by several authors in the literature.^5^ ^,^ ^9^ ^,^ ^19^ It is believed that fetal hemoglobin has a greater affinity to oxygen than does adult hemoglobin. Thus, a transfusion of adult hemoglobin could generate possible hyperoxia due to increased oxygen delivery to tissues.^27^ Another theory is that there could be an increase in free radicals after the transfusions due to an increase in plasma free iron.^28^ ^-^ ^30^

Logistic regression takes into account the relative contributions of various factors and makes it clear that the true risk factor is low gestational age and the consequential immaturity of the premature infant's various tissues. In other words, the true risk factor is prematurity and the other factors (low SNAPPE II score at birth, bronchopulmonary dysplasia, use of oxygen in mechanical ventilation, use of diuretics and the necessity for blood transfusions) are just consequences of this prematurity and are driven towards the same statistical significance observed in isolated tests. Healthcare professionals should be alert to the risk of ROP among preterm infants, and such awareness should guide preventive and timely care. Further studies should take into consideration the risk factors identified in this study, as well as the possibility of new variables that imply risks for premature infants.

## CONCLUSIONS

The observed incidence was higher than that found in the literature, thus showing that occurrences of retinopathy of prematurity remain high among infants with very low birth weight. The development of ROP was inversely proportional to the weight and gestational age at birth. Prevention of prematurity and caution in using oxygen in neonatal intensive care units may help reduce the future incidence of retinopathy of prematurity.
